# Phenotypic expression of swallowing function in Niemann–Pick disease type C1

**DOI:** 10.1186/s13023-022-02472-w

**Published:** 2022-09-05

**Authors:** Beth I. Solomon, Andrea M. Muñoz, Ninet Sinaii, Nicole M. Farhat, Andrew C. Smith, Simona Bianconi, An Dang Do, Michael C. Backman, Leonza Machielse, Forbes D. Porter

**Affiliations:** 1 Speech-Language Pathology Section, Mark O. Hatfield Clinical Center, National Institutes of Health, 9000 Rockville Pike, Bld. 10 1-NW-1455, Bethesda, MD 20892 USA; 2 Eunice Kennedy Shriver National Institute of Child Health and Human Development, National Institutes of Health, Bethesda, MD USA; 3 Biostatistics and Clinical Epidemiology Service, NIH Clinical Center, National Institutes of Health, Bethesda, USA; 4 Biostatician, Silver Spring, MD USA

## Abstract

**Background:**

Niemann–Pick disease type C1 (NPC1) is a rare autosomal recessive disease characterized by endolysosomal accumulation of unesterified cholesterol with progressive deterioration in swallowing, often leading to premature death. Although documented, the natural history of NPC1 swallowing dysfunction has yet to be delineated systematically. This manuscript aims to provide a comprehensive characterization of the phenotypic spectrum and progression of swallowing dysfunction in NPC1.

**Methodology:**

The National Institutes of Health (NIH) NPC1 natural history study (NCT00344331) enrolled 120 patients, who underwent comprehensive interpretative swallow assessments for swallowing safety, dietary modifications, and aspiration risk. Longitudinal statistical modeling accounted for all outcomes with NPC1 disease covariates (first symptom onset, age at neurological symptom onset, seizure history, duration of neurological symptoms) as well as miglustat use (a glucosylceramide synthase inhibitor) and NIH study duration (NIHSD; the length of time an individual participated in the NIH study). Probabilities for disease progression and time to swallowing decline were conducted for the entire cohort.

**Results:**

Time to swallowing decline with American Speech-Language-Hearing Association National Outcome Measure (ASHA-NOMS) and the NIH-adapted Penetration Aspiration Scale (NIH-PAS) were identified: $\frac{7.7}{100}$ person-years and $\frac{9.8}{100}$ person-years, respectively. NIHSD and seizure history consistently and significantly were associated with decline (*OR*_*NIHSD*_ = 1.34–2.10, 95% *CI* 1.04–3.4, *p* = 0.001–0.026; *OR*_*Seizure*_ = 3.26–18.22, 1.03–167.79; *p* = 0.001–0.046), while miglustat use revealed protection (*OR*_*Miglustat*_ = 0.01–0.43, 0.007–0.98; *p* = 0.001–0.044). The probability of decline with NPC1 neurological severity scale and annual severity increment scale were established with the aforementioned covariates, varying amongst subgroups.

**Conclusion:**

This study represents the most extensive collection of prospective, instrumental swallowing assessments in NPC1 to date with an interpretive analysis providing an improved understanding of NPC1 disease progression with swallowing function—serving as a foundation for clinical management and future NPC1 therapeutics.

**Supplementary Information:**

The online version contains supplementary material available at 10.1186/s13023-022-02472-w.

## Background

With an estimated incidence of 1/100,000 live births, Niemann–Pick disease type C1 (NPC1; OMIM:257220) is a rare autosomal recessive disorder characterized by the endolysosomal accumulation of unesterified cholesterol due to a defect in intracellular cholesterol trafficking [1]. Loss-of-function variants in either *NPC*1 (~ 95% of cases) or *NPC2* result in an accumulation of cholesterol in the central nervous system and peripheral organs, resulting in NPC1 visceral and neurologic phenotypes [2]. NPC1 is a fatal, pediatric neurodegenerative disease with symptoms including cerebellar ataxia, vertical gaze palsy, dysarthria, and dysphagia, which occurs in 55–80% of affected individuals [3, 4]. As dysphagia a significant morbidity within NPC1, this paper aims to provide a comprehensive characterization of the phenotypic spectrum and progression of swallowing dysfunction within the National Institutes of Health (NIH) NPC1 cohort.

*Oropharyngeal dysphagia* often referred to as abnormal swallowing function within the oral cavity, pharynx, and larynx—consists of a synchronized series of maneuvers with > 30 paired muscles and numerous cranial nerves under cortical and brain stem control [5]. Any dyscoordination/physiologic variation can result in dysfunction, possibly resulting in *laryngeal penetration* or *aspiration*. Laryngeal penetration refers to the entry of food, liquid, or oral secretions into the laryngeal vestibule without passage through the true vocal folds [5]. Conversely, aspiration refers to the passage below the vocal folds into the tracheobronchial airway [5]. Either event may involve an overt, protective clearance response (e.g. coughing); however, this sensory-motor reaction tends to diminish over time resulting in *silent aspiration* [6]. Silent aspiration is defined by the entry of any foreign substances entering the upper airway without signs of coughing/choking [6]. Although non-specific to NPC1, the prevalence of silent aspiration is suggested to be > 40% in patients with progressive neuromuscular diseases [6]. Without direct visualization, silent aspiration may be missed in clinical swallow examinations. To adequately assess physiologic swallowing functions and aspiration risks, an instrumental assessment is needed.

Although aspiration is regarded as a pivotal dysphagia event, other coexisting physiological impairments (e.g., movement dyscoordination, weakened muscular contraction, and hypopharyngeal retention) may be equally worrisome. Consequently, dysphagia is an important risk factor for NPC1 morbidity, and likely mortality as it frequently results in aspiration pneumonia and other respiratory comorbidities [4]. Several therapeutic trials have attempted to utilize swallowing as an outcome measure; however, a prospective natural history of swallowing function has yet to be reported [7, 8, 9]. NPC1 clinical trial design is further complicated by the rarity of this disorder and significant genetic and phenotypic heterogeneity amongst patients. Any therapeutic trial would significantly benefit from longitudinal data on NPC1 swallowing dysfunction.

## Methods

### Population demographics

**Fig. 1:**
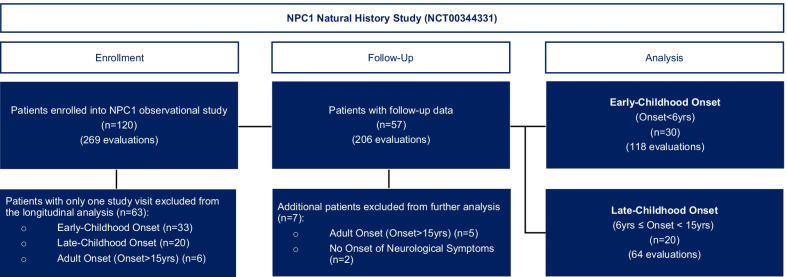
CONSORT diagram of patient enrollment in National Institutes of Health Niemann–Pick disease Type C1 (NPC1) natural history study (NCT00344331)

Between 1997 and 2019, 120 patients with a confirmed diagnosis of NPC1 were enrolled under an NIH Internal Review Board (IRB)-approved NPC1 natural history study (NCT00344331) (Fig. 1). Patients or guardians provided consent/assent. This study was initiated to identify clinical or biochemical markers that could be utilized as outcome measures. As swallowing dysfunction is a known NPC1 morbidity, the NIH study included the following evaluations: history, physical, cranial nerve [10] (oral-motor assessments), swallowing with videofluoroscopic swallowing studies (VFSS) when clinically indicated, and speech assessments. All subjects were assessed at baseline, and 57 (47.5%) patients were followed longitudinally over a median of 3.3 years [interquartile ratio (IQR): 1.3–4.4; range 0.6–10.3] and median 4.0 visits (IQR: 3.0–6.0; range 2.0–11.0) for a cumulative study total of 206 evaluations. These 57 patients were categorized into Early-Childhood Onset (ECO, neurological onset < 6 years), Late-Childhood Onset (LCO, neurological onset ≥ 6 years to < 15 years), Adult Onset (AO, neurological onset > 15 years), and those with no onset of neurological symptoms subgroups. Yearly follow-up was attempted; however, this study experienced attrition and varying temporal lengths between visits accounted for in the longitudinal models (Additional file 1: Fig. S1).

### NPC1 history, physical, and neurological assessments

We obtained the following parameters from patient history: age at first NPC1 symptom onset, age at neurological symptom onset, age at NPC1 diagnosis, seizure history, and miglustat use. First symptom onset was classified by any NPC1-specific symptoms, including psychiatric disorders [11] such as schizophrenia and persecutory hallucinations (typically seen in AO patients) and peripheral diseases such as hepatosplenomegaly and jaundice. In contrast, neurological disease onset was categorized by clinical symptoms that originated from the central nervous system. The NPC1 neurological severity scale (NSS), a clinical outcome measure, was scored as described by Yanjanin et al. in 2010 and consists of disease-specific domains including swallow, speech, fine motor, cognition, ambulation, and memory [12]. NSS_Motor_ (NSS_Ambulation_ + NSS_FineMotor_) and NSS_Cognitive_ (NSS_Cognition_ + NSS_Memory_) were computed for secondary analysis. The annual severity increment score (ASIS) was calculated to quantify age-adjusted disease severity [13].

### Oral-motor and speech examinations

An NIH speech-language pathologist (SLP) conducted comprehensive oral-motor exams assessing tongue and lip strength deficits in isolated and alternating movement tasks. The extent of dysarthria—defined by the American Speech-Language-Hearing Association (ASHA) as a speech disorder caused by muscle weakness—was rated from conversational samples assessing global parameters of articulation, rate, prosody, and vocal quality (Table 1) [14].

**Table 1:**
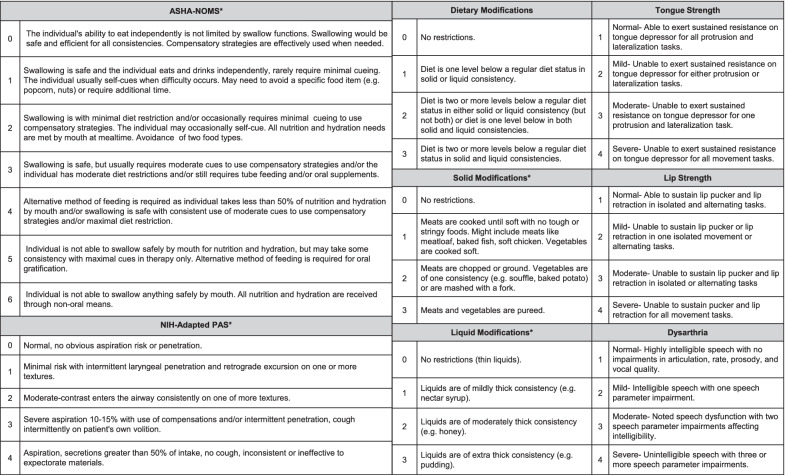
Videofluoroscopic swallow study (VFSS) primary outcome measures

*ASHA-NOMS* American Speech-Language-Hearing Association National Outcome Measures Scale, *NIH-PAS* NIH-adapted penetration and aspiration scale

^*^Scales were reversed and/or transformed to make them consistent with the direction of all rating scales used in the analysis

### Swallowing examinations

Patients were screened via interview and questionnaire to determine the presence of dysphagia and the need for a formal instrumental evaluation. A patient’s medical history along with proxy-reported swallowing dysfunction including intermittent coughing or choking when eating/drinking, difficulty chewing hard texture foods, and a sensation of food sticking in the back of the mouth or throat facilitated the need for instrumental assessments. VFSS was systematically assessed with liquid, puree, and solid textures to determine swallowing physiology for safe nutritional intake. Swallowing was assessed utilizing 26 impairment-level parameters of oropharyngeal function (Additional file 1: Table S1) [15]. Based on clinical VFSS findings, the SLP determined appropriate clinical management.

### Interpretative VFSS analysis

Frequently employed as an SLP treatment outcome measure, the ASHA National Outcomes Measurement System (ASHA-NOMS) was applied as an interpretive summary rating scale for VFSS [16]. The ASHA-NOMS swallowing domain and dietary modification subdomains were applied to interpret overall swallowing ability and safety [16]. Additionally, we adapted the Rosenbek Penetration/Aspiration Scale (NIH-PAS) to facilitate clinical research interpretations for aspiration risk identification [5]. The NIH-adapted PAS was used to determine swallowing safety and quantify the extent of aspiration/laryngeal penetration [5]. Each scale independently (1) provides valuable data for clinical management and research, (2) tracks swallow outcomes longitudinally, and (3) follows changes with other disease covariates. Scales for ASHA-NOMS, NIH-PAS, solid modifications, and liquid modifications were reversed to match the direction of increasing severity of the other scales and were analyzed accordingly [14].

### Statistics

Data are described using frequency (percentage), mean (± standard deviation), or median (inter-quartile range). Comparisons amongst or between groups used analysis of variance or Kruskal–Wallis tests, as appropriate, with stepdown Bonferroni adjusted posthoc pairwise comparisons or Wilcoxon rank-sum tests. Fisher’s exact tests compared categorical data and ordinal data by Cochran–Mantel–Haenszel row mean scores. Correlations utilized Spearman’s rho. Longitudinal analyses of data were performed in the overall cohort, ECO and LCO subgroups, using generalized linear models for repeated measures with independent working correlations for ordinal multinomial and continuous models (SAS Institute, Inc, Cary, NC). The longitudinal models excluded the AO subgroup and those who had not yet developed neurological manifestations. Probabilities for the severity of disease were modeled for primary (ASHA-NOMS, NIH-PAS, dietary, liquid, and solid modifications, tongue and lip strength, and dysarthria) and secondary (NSS_Total_, NSS_Swallow_, NSS_Motor_, NSS_Cognitive_, and ASIS) outcomes. Models accounted for the effects of NIH study duration (NIHSD), representing the length of time that an individual participated in the NIH study with/without neurological disease onset and can serve as a proxy for swallowing dysfunction progression. Additionally, the duration of neurological symptoms, seizure history, and miglustat use were included in the modeling. Majority of our cohort was prescribed miglustat for off-label use as it is not approved United States Food and Drug Administration (FDA) for NPC1. In these longitudinal models, the important disease aspects of neurological onset (ECO and LCO subgroups as fixed effects) and duration of neurological symptoms (as a random effect) were captured as complementary factors. Time to swallowing decline by ASHA-NOMS and NIH-PAS was estimated using the Kaplan–Meier survival function and described as a median [95% confidence intervals (CI)]; differences in survival distributions between ECO and LCO were compared using the log-rank test.

## Results

### Demographics

Of the 120 NPC1 patients, 114 (95%) presented with neurological manifestations; 63 (52.5%) ECO, 40 (33.3%) LCO, and 11 (9.2%) AO (Table 2). The remaining 6 (5.0%) had yet to develop neurological signs or symptoms. As expected, neurologically affected subgroups differed by the median age at initial visit (*p* < 0.001), the median age at neurological disease onset (*p* < 0.001), the duration of neurological symptoms (*p* < 0.001), and the median age at first symptom onset (*p* < 0.001). In some cases, the age at first symptom onset was the same age at first neurological symptom onset. The ECO subgroup demonstrated a shorter diagnostic delay compared to the LCO (*p* < 0.001) and AO (*p* = 0.001) subgroups. At baseline, the subgroups had no differences in sex, presence of seizures, or miglustat use. The mean age of seizure onset in the ECO subgroup (6.3 ± 4.2 years) was lower than LCO (11.9 ± 4.4 years, *p* = 0.014) subgroup. Only one AO patient experienced seizures with onset at 18.0 years. Upon baseline visits, patients generally exhibited normal swallowing function in all outcomes and subgroups (Table 2).

**Table 2:** Demographic, clinical characteristics, and primary outcomes of patients with Niemann–Pick disease Type C1 (NPC1) at baseline

Characteristic	All patients (n = 120)	Early childhood (n = 63)	Late childhood (n = 40)	Adult onset (n = 11)	No neurological symptoms (n = 6)	Overall *p* value^a^	ECO versus LCO *p* value^b^
Demographic							
Age at visit, *median (IQR), years*	9.8 (3.8–19.0)	4.5 (2.9–8.5)	17.3 (12.6–21.1)	36.1 (32.5–62.5)	1.5 (1.1–9.7)	< 0.001	< 0.001
Sex, F	63 (52.5%)	29 (46.0%)	21 (52.5%)	9 (81.8%)	4 (66.7%)	0.15	0.55
Patients with follow-up visits, *(n* = *57)*	57 (100%)	30 (52.6%)	20 (35.1%)	5 (8.8%)	2 (3.5%)	0.66	0.98
Clinical							
Onset of neurological symptoms^c^	114 (95.0%)	63 (100.0%)	40 (100.0%)	11 (100.0%)	0 (0.0%)	< 0.001	NA
Age at onset of neurological symptoms*, median (IQR), years*	5.0 (2.0–8.0) (n = 114)	2.0 (1.5–3.0)	8.0 (6.5–11.0)	21.0 (18.0–43.0)	NA	< 0.001	< 0.001
Duration of neurological symptoms, *median (IQR), years*	4.2 (1.2–10.3) (n = 114)	2.0 (0.3–5.4)	8.6 (4.5–11.8)	15.9 (10.5–17.9)	NA	< 0.001	< 0.001
Onset of seizure symptoms	29 (24.2%)	20 (31.8%)	8 (20.0%)	1 (9.1%)	0 (0.0%)	0.18	0.26
Age at onset of seizures, *mean* (*SD*)	8.2 (5.2) (n = 29)	6.3 (4.2)	11.9 (4.4)	18 (.)	NA	0.003	0.014
Seizure diagnosis	16 (13.3%)	10 (15.9%)	5 (12.5%)	1 (9.1%)	0 (0.0%)	0.90	0.78
Age at onset of first symptom, *median (IQR), years*	1.1 (0–6.0)	0.1 (0–1.5)	6.0 (1.5–8.5)	21.0 (18.0–43.0)	0 (− 0.3 to − 3.0)	< 0.001	< 0.001
Duration of diagnostic delay, *median (IQR), years*	4.0 (1.0–9.0) (n = 119)	1.9 (0.7–4.5) (n = 62)	8.5 (4.5–11.5)	11.0 (5.0–17.0)	0.7 (0.3–9.0)	< 0.001	< 0.001
Miglustat use	44 (36.7%)	21 (33.3%)	19 (47.5%)	4 (36.4%)	0 (0.0%)	0.13	0.21
Primary outcomes							
ASHA-NOMS, *median score (IQR)*	0 (0–0)	0 (0–0)	0 (0–1.0)	0 (0–1.0)	0 (0–0)	0.18	0.20
PAS, *median score (IQR)*	0 (0–0)	0 (0–0)	0 (0–0.5)	0 (0–1.0)	0 (0–0)	0.085	0.21
Dietary modification, *median score (IQR)*	0 (0–0)	0 (0–0)	1.0 (0–1.0)	0 (0–1.0)	0 (0–0)	0.003	0.009
Solid modification, *median score (IQR)*	0 (0–0)	0 (0–0)	0 (0–0.5)	0 (0–0)	0 (0–0)	0.34	0.17
Liquid modification, *median score (IQR)*	0 (0–0)	0 (0–0)	0 (0–1.0)	0 (0–1.0)	0 (0–0)	0.095	0.16
Tongue strength, *median score (IQR)*	2.0 (1.0–3.0) (n = 115)	2.0 (1.0–3.0) (n = 60)	2.0 (2.0–3.0)	3.0 (2.0–3.0)	1.0 (1.0–1.5) (n = 4)	0.036	0.21
Lip strength, *median score (IQR)*	2.0 (1.0–3.0) (n = 114)	2.0 (1.0–3.0) (n = 60)	2.0 (1.0–3.0) (n = 39)	3.0 (2.0–3.0)	1.0 (1.0–1.0) (n = 4)	0.011	0.11
Dysarthria, *median score (IQR)*	2.0 (1.0–3.0) (n = 108)	2.0 (1.0–3.0) (n = 56)	3.0 (2.0–3.0) (n = 38)	3.0 (2.0–3.0)	1.0 (1.0–1.0) (n = 3)	0.008	0.051

Data are n (%), unless otherwise specified. Percentages may not total 100% due to rounding. Where data were missing, the available n is shown

*ECO* Early-Childhood Onset (< 6 years old), *LCO* Late-Childhood Onset (≥ 6 to < 15 years old), *AO* Adult Onset (≥ 15 years old), *SD* standard deviation, *IQR* inter-quartile (25th–75th percentile) range, *NA* not applicable

^a^*p* value from testing the global null hypothesis using ANOVA or Kruskal–Wallis test, as appropriate, or Fisher's exact test

^b^*p* value from stepdown Bonferroni adjusted post-hoc pairwise comparison or Wilcoxon rank sum test, as appropriate, or Fisher's exact test

^c^Onset of neurological symptoms was categorized as clinical symptoms that originated from the central nervous system

### Time to swallowing decline

**Fig. 2:**
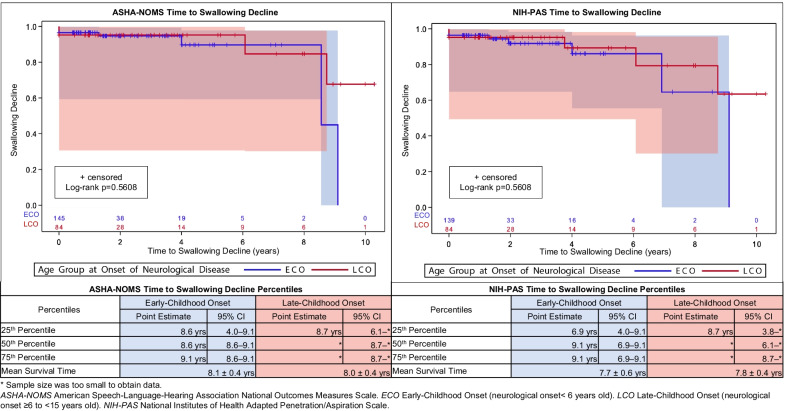
Niemann–Pick disease Type C1 (NPC1) time to swallowing decline survival plots for the American Speech-Language-Hearing Association National Outcome Measures Scale (ASHA-NOMS) and the NIH-Adapted Rosenbek Penetration and Aspiration Scale (NIH-PAS)

Our objective was to identify a specified level in both interpretive scales where decline occurred, but due to NPC1 disease heterogeneity, this was unfeasible. Therefore, for clinical purposes, we defined swallowing decline as the time-point when oral nutrition was < 50% by mouth requiring alternate feeding methods (ASHA-NOMS ≥ 4), and moderate aspiration was noted with at least one or more textures (NIH-PAS ≥ 2). For ASHA-NOMS, the incidence rate was $\frac{7.7}{100}$ person-years, the mean age of swallowing decline was 14.9 ± 8.8 years, and the mean duration of neurological symptom was 10.0 ± 6.6 years. For NIH-PAS, the incidence rate was $\frac{9.8}{100}$ person-years, the mean age of swallowing decline was 16.5 ± 8.4 years, and the mean duration of neurological symptoms to swallowing decline was 11.1 ± 5.5 years. The mean time from baseline to decline was 2.5 ± 3.7 years for ASHA-NOMS and 2.3 ± 3.3 years for NIH-PAS. Interestingly, time to swallowing decline for both primary outcomes did not differ between ECO and LCO (Fig. 2). The patterns for swallow decline progression were heterogeneous for primary outcomes without any trends identified nor improvement once the threshold was met.

### Primary outcomes

**Fig. 3:**
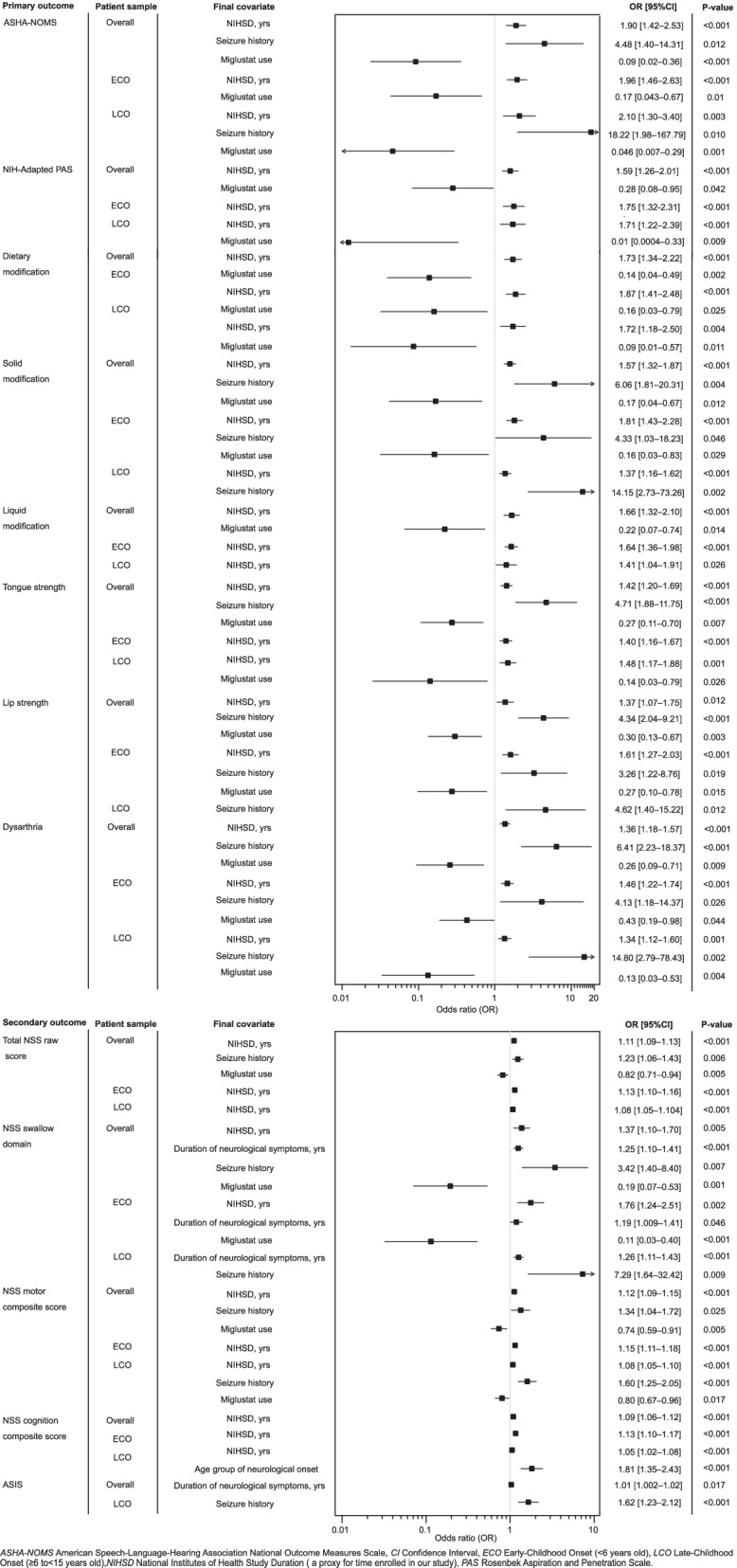
Forest plots of longitudinal analysis results of all outcomes in patients with Niemann–Pick disease Type C1

NPC1 outcomes analyses were performed to interpret VFSS data to determine safe swallowing function, clinical management, and aspiration risk (Fig. 3). Additionally, supplemental analyses of dietary, solid, and liquid modifications, tongue and lip strengths, and dysarthria were performed to investigate their associations on swallowing for clinical management. Modeling suggested worsening ASHA-NOMS and NIH-PAS scores with longer NIHSD across all groups. Seizure history was also associated with worsening ASHA-NOMS scores in the overall cohort and LCO subgroup. As expected with a progressive disease, the models demonstrated an increased risk for worsening modification scores amongst all groups with NIHSD. Conversely, miglustat use demonstrated a protective effect amongst all groups for ASHA-NOMS, while only for the entire cohort and LCO subgroup for NIH-PAS, previously reported [16]. Miglustat use also revealed a protective effect across all subgroups for dietary modifications, but only for the overall group for solid and liquid modifications.

Oral-motor assessments of tongue and lip strengths were conducted to associate possible physiologic correlates within oropharyngeal swallowing (Fig. 3). Our models demonstrated an increased risk for diminished tongue strength in all groups with NIHSD; however, this was only observed in the overall cohort and the ECO subgroup for lip strength. Furthermore, seizure history increased the risk for diminished tongue strength only in the overall cohort. An increased risk for weakened lip strength was identified with seizure history across all groups. Miglustat use demonstrated a protective effect for tongue and lip strengths in the overall cohort, although not consistent amongst the subgroups.

Dysarthria—weakened speech production associated with diminished lip and tongue strengths—is a speech correlate to swallowing dysfunction [14]. Higher NIHSD and a history of seizures increased the risk for dysarthria in all groups, whereas miglustat was protective (Fig. 3).

### Secondary outcomes

We utilized multiple measures in the natural history study to determine and phenotype NPC1. Our extensive analysis utilized NSS (NSS_Total_, NSS_Swallow_, NSS_Motor_, and NSS_Cognitive_) and ASIS to determine relationships with the covariates (Fig. 3). This analysis was intended to assess covariates associated with specified domains linked to functional activities. The NSS is utilized clinically and in research to correlate clinical data with disease severity and progression [12]. Longitudinal analysis suggested similar results as above, with increased risk for a worsening NSS_Total_ score with NIHSD in the entire cohort and subgroups. Seizure history was also associated with a worsening NSS_Total_ score in the overall cohort. Contrarily, miglustat use revealed a protective effect. As swallowing is an important NPC1 characteristic, we utilized the NSS_Swallow_ domain to evaluate our covariates further. We identified an increased risk for worsening swallow reports for many covariates in the entire cohort with varying results amongst subgroups. Furthermore, NSS_Swallow_ demonstrated a significant correlation (ASHA-NOMS r_s_ = 0.54, NIH-PAS r_s_ = 0.54; *p* < 0.001 for both). NSS composite scores were derived to further phenotype NPC1 functional outcomes with the covariates. An increased risk of a worsening NSS_Motor_ score with NIHSD was observed consistently across all groups. Additionally, seizure history demonstrated an increased risk of a worsening NSS_Motor_ score in the overall cohort and LCO subgroup. Miglustat use demonstrated a protective effect for composite scores in the entire cohort and LCO subgroup. Only NIHSD is associated with a worsening NSS_Cognitive_ score amongst all groups.

ASIS, a metric for clinical monitoring and a possible therapeutic outcome, was also analyzed (Fig. 3). ASIS scores were higher in the ECO subgroup than the LCO subgroup in the overall cohort (*p* < 0.001). The duration of neurological symptoms and seizure history increased the risk of a worsening ASIS score within the LCO subgroup.

### Supplemental outcomes

Silent aspiration is a concerning respiratory comorbidity, commonly unidentified in clinical swallowing exams without VFSS. We identified that 12.4% of all our evaluations exhibited silent aspiration, resulting in immediate clinical management such as dietary modifications and interventions. We recommend gastrostomy tube (G-tube) placement as a supplemental/alternate means of nutrition while maintaining some safe oral modifications for gratification when patients exhibited ASHA-NOMS scores ≥ 4 or NIH-PAS scores ≥ 2. Subsequently, 17% (n = 20) of our patients at baseline and 16% (n = 19) at follow-up utilized G-tubes. Of these patients, the median time G-tube placement occurred was at 10.5 years (range 1.5–22 years) from neurological symptom onset.

## Discussion

Dysphagia is a primary concern for NPC1 patients and families because of its safety and social implications [12]. Although documented throughout the literature, the understanding of NPC1 swallowing progression is limited with only a few studies applying systematic instrumental assessments to visualize swallowing safety and aspiration risks [7, 8, 9, 17]. We acknowledge that our interpretive rating scales may not be widely accessible or recognized by other health care providers. However, these tools offer a summative interpretive analysis guiding clinical management and treatment outcome, unlike other methodologies in NPC1 literature [5, 18]. Our report further expands upon the NPC1 phenotype to include swallowing safety, need for diet modifications, laryngeal penetration, aspiration risks, and other speech domains, which have not been addressed comprehensively in the literature.

This large research data collection for the ultra-rare disease of NPC1 is unique and difficult to conduct due to limited patient cohort, disease progression over time, and lack of suitable control groups. We acknowledge the lack of severely affected NPC1 patients in our study, possibly impacted by travel ability. The AO subgroup was limited and may be attributed to varying clinical presentations or misdiagnosis of similar neuro-progressive disorders. Although comparable, the ECO and LCO subgroups differed slightly in the number of patients, which may have impacted subgroup analysis.

In the NIH NPC1 natural history cohort, we confirmed that swallowing function in NPC1 declined over time. Despite differences between groupings in cognitive function [19], there were no differences between ECO and LCO subgroups for swallowing outcomes. We also established covariates with confirmed associations with known NPC1 parameters affecting swallowing decline.

### Swallowing outcomes

#### Time to swallowing decline

There is limited NPC1 literature regarding the time to swallowing decline. Unfortunately, our investigation was unsuccessful in identifying a consistent level of decline using the interpretive measures; therefore, our clinical experience determined specific levels of swallowing decline. NPC1 disease heterogeneity may explain the wide range in time until swallowing decline from neurological disease onset and the duration of neurological symptoms. Among NPC1 providers, the clinical presentation in swallowing function may appear worse due to earlier presentations in neurologic manifestations; however, no differences in time to swallowing decline were identified between subgroups. This is likely attributed to differences in sample size. Once the threshold of swallowing decline was met, a nonlinear trend without improvement was observed. This may be related to NPC1 disease progression, our study’s attrition, and varying temporal lengths in follow-up.

#### Primary outcomes analysis

This study confirmed the successful application of ASHA-NOMS and NIH-PAS, highlighting swallowing safety and aspiration risk within our cohort. Similar to the NPC1 disease heterogeneity, there are potentially many underpinnings contributing to the swallowing dysfunction: cerebellar ataxia [20], cranial nerve dysfunction causing oral-motor weakness [21], and dyscoordination of swallowing and respiratory cycles [22, 23]. Our in-depth analysis has revealed three variables (NIHSD, seizure history and miglustat use) consistently and significantly affecting swallowing physiology outcomes.

Worsening swallowing function was consistently associated with NIHSD throughout our NPC1 cohort. We anticipated this variable’s significance as functional deterioration occurs in neuro-progressive diseases over time. Additionally, diminished lip strength with NIHSD demonstrated variability amongst subgroups, despite functional lip closure in all patients. Heterogeneity and differences in subgroup size may explain this finding.

A history of seizures, another known NPC1 comorbidity, was associated with an increased risk of worsening swallowing function among oral-motor and speech parameters [3]. This was nonsignificant for worsening NIH-PAS scores, likely related to the reduced number of severity rating domains within the scale. We expected dietary modifications amongst our cohort due to possible NPC1 cerebellum dysfunction affecting motor coordination and bolus transit with liquids [24]. However, unanticipated, solid modifications were identified consistently likely related to tongue strength deficits impacting bolus motility. We suggest that the increased risk of swallowing decline with seizures is multifactorial (dyscoordination, seizure polypharmacy, fatigue, and NPC1 heterogeneity). Additionally, we acknowledge that seizure medications can also impact swallowing ability, affecting cognitive/motor responses [25, 26, 27, 28]. Furthermore, a history of seizures and oral-motor weaknesses can contribute to the observed NPC1 dysarthria. Although we may not have a clear understanding of the role of NPC1 disease deterioration on seizure history and worsening speech/swallowing function, we demonstrated there is a significant association**.** Study limitations include limited documentation of seizure medications, seizure categorization, and pharmacological controls. Although not approved by the United States FDA for NPC1, 37% of our cohort received off-label miglustat therapy at baseline. Therefore, accounting for its effect was necessary. Previously published, miglustat use revealed a protective effect with ASHA-NOMS and NIH-PAS [29, 30, 31]. However, in this paper, we confirm its same effect with additional primary outcomes. Variability amongst subgroups may be attributed to the timing of initial miglustat treatment, cumulative miglustat dosage, and NPC1 disease heterogeneity. Perhaps miglustat’s known mechanism of action, inhibiting glucosylceramide synthase [32], may also contribute to the preservation of swallowing, speech, and oral-motor functions, further supporting its clinical use [29].

#### Secondary outcomes analysis

We confirmed the risks of decline in swallowing function with our NPC1 phenotypic parameters, utilizing natural history study data domains (NSS_Total_, NSS_Swallow,_ NSS_Motor_, NSS_Cognitive_, and ASIS). The significant and consistent variables noted above with our primary outcomes were also revealed with the secondary outcomes. NSS composite scores served as prerequisites of motor function and cognitive awareness needed for swallowing and speech to further phenotype NPC1, similar to others who utilized severity scores (e.g. Clinical Severity Scale, NSS) to document disease progression [33, 34, 35]. The subgroup and covariate variability among NSS scores may be attributed to reasons like those previously mentioned with the primary outcomes and parental/caregiver report biases (NSS) and silent aspiration (NSS_Swallow_). The NSS provides a unique account of daily functioning, often not captured in clinical performance assessments. Furthermore, the NSS_Swallow_ domain correlations with ASHA-NOMS and NIH-PAS provide additional support for the combined use of parent-reported outcomes with instrumental swallow assessments.

ASIS, a proxy of disease progression and severity [13], also confirmed the increased risk of swallowing decline with an earlier age of neurological onset in the overall cohort. This was an expected finding because our ECO patients developed more neurological symptoms since initial enrollment compared to the LCO subgroup—resulting in an increased disease burden. NPC1 disease heterogeneity likely influenced the variability in covariates amongst groupings. Of note, we excluded the duration of neurological disease from our model as the calculation includes chronological age and the duration of neurological disease—potential confounders. Although ASIS scores can be informative on an individual basis, it is important to understand this value’s meaning when comparing scores within patients, between age-groupings, and within statistical modeling due to its computational biases.

#### Supplemental outcomes analysis

Despite G-tube advisement when clinically indicated, the data may not accurately represent G-tube due to family non-compliance. We underscore that swallowing decline is variable and gradual, thus this outcome measure may not be applicable for short-term therapeutic trials.

### NPC1 dysphagia management

This study provides insight for clinical practice, underscoring specific disease parameters and incidence of swallowing decline for management. Our clinical expertise and NPC1 phenotype findings offer a framework to support swallowing safety with the following patient-care recommendations:

- NPC1 patients should have a baseline swallowing assessment upon diagnosis by an SLP with yearly reassessments—VFSS should be considered when clinically indicated.
- Education should be provided to patients and families regarding swallowing function, aspiration precautions, and management strategies.
- Patients and families should adhere to SLP-recommended therapeutic protective maneuvers, positioning, utensil modifications, and alternative feeding methods—in home, education, and social settings.

These management recommendations agree with and expand upon the NPC clinical consensus guidelines, highlighting the possible need for alternate feeding methods with disease progression [28].

## Conclusion

Our study represents the most extensive collection of prospective, instrumental swallowing assessments in NPC1 to date. This large data series can serve as a foundation for NPC1 swallowing and speech domains assisting clinical management, quality of life decisions, and therapeutic outcomes for future clinical trials. Utilizing VFSS to visualize swallowing physiology directly, we identified NPC1 swallowing impairments, including silent aspiration often missed in clinical swallowing examinations. Thus, an interpretive quantitative analysis of VFSS was applied to phenotype NPC1 swallowing physiology and aspiration risk, facilitating clinical management. We observed a non-linear, heterogeneous rate of swallowing decline with the primary outcomes in our cohort. Additionally, the covariates of seizure history and NIHSD were observed to significantly increase the risk of swallowing decline, while miglustat use demonstrated a protective effect—previously identified in the literature [29]. This study revealed miglustat’s protection of swallowing function and its positive effects on speech and oral-motor functions, encouraging its clinical use among NPC1 patients.

Our interpretive analysis and statistical modeling from the natural history study provide an improved understanding of NPC1 disease progression with swallowing function. Additionally, we conclude that these swallowing assessments with this interpretive analysis should be considered for other rare disease research.

## Supplementary Information

**Additional file 1: Fig. S1.** National Institutes of Health Niemann–Pick disease Type C1 patient timelines and longitudinal follow-up. **Table S1.** Videofluoroscopic swallow study (VFSS) swallowing impairments assessed.

## Data Availability

De-identified data can be made available with approved research agreements with the National Institutes of Health. Beth I. Solomon, the corresponding author, accepts full responsibility for this publication.

## Abbreviations

ASHA: American Speech-Language-Hearing Association
ASHA-NOMS: American Speech-Language-Hearing Association National Outcomes Measurement System
ASIS: Annual severity increment score
AO: Adult onset
CI: Confidence interval
ECO: Early-childhood onset
FDA: Food and Drug Administration
G-tube: Gastrostomy tube
IQR: Interquartile ratio
IRB: Internal Review Board
LCO: Late-childhood onset
NIH: National Institutes of Health
NIHSD: National institutes of health study duration
NPC1: Niemann–Pick disease type C1
NSS: Niemann–Pick disease type C1 neurological severity scale
OR: Odds ratio
NIH-PAS: National institutes of health penetration/aspiration scale
SLP: Speech-language pathologist
VFSS: Videofluoroscopic swallowing studies
