# Supplementary material for: Phenotypic expression of swallowing function in Niemann–Pick disease type C1
Source: Orphanet J Rare Dis. 2022 Sep 5;17:342. doi: 10.1186/s13023-022-02472-w (PMC9446530; doi:10.1186/s13023-022-02472-w)
Supplement: Supplementary file 1 — Additional file 1: Fig. S1. National Institutes of Health Niemann–Pick disease Type C1 patient timelines and longitudinal follow-up. Table S1. Videofluoroscopic swallow study (VFSS) swallowing impairments assessed. [file 13023_2022_2472_MOESM1_ESM.pptx]

## Slide 1
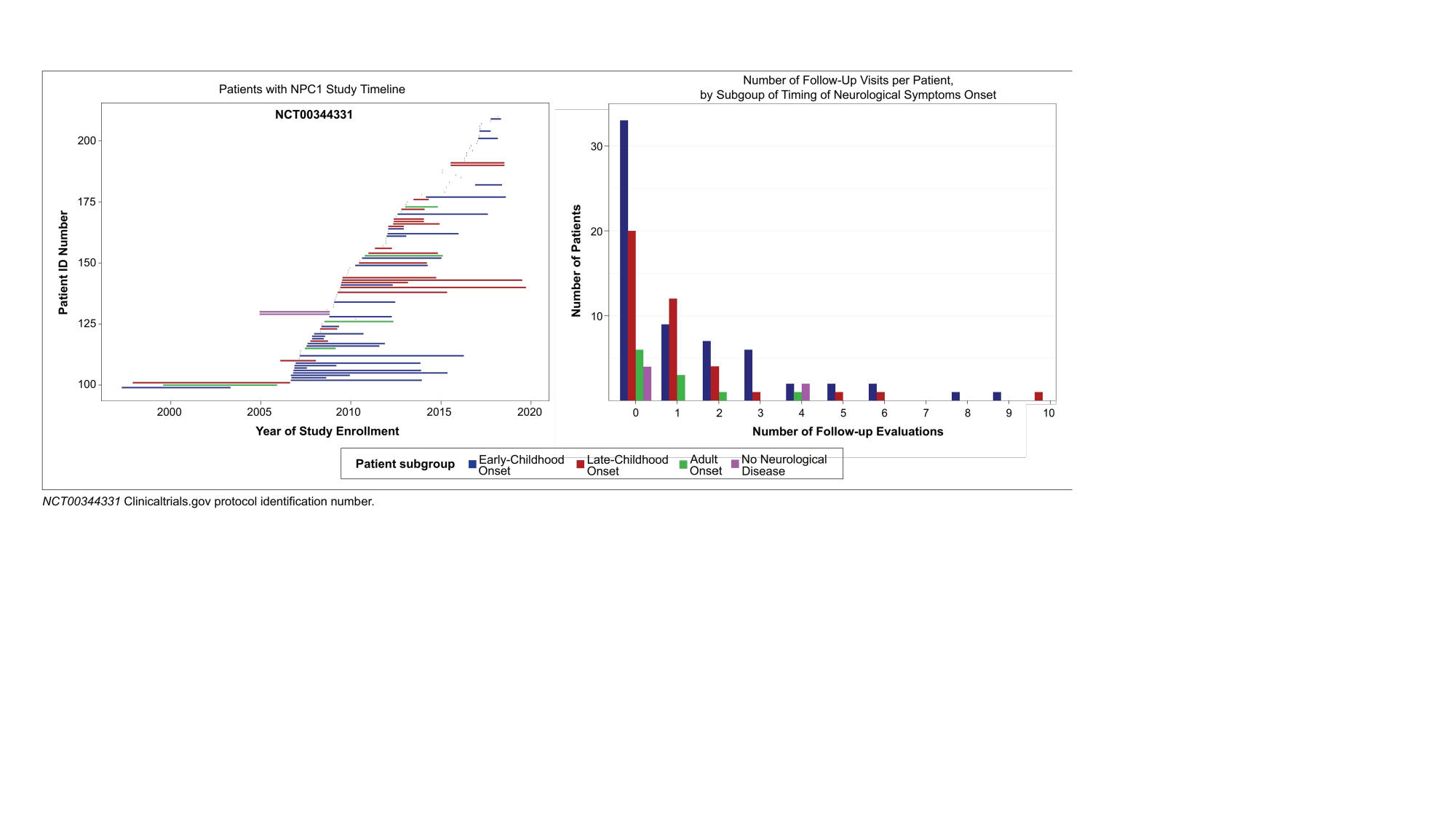

## Slide 2
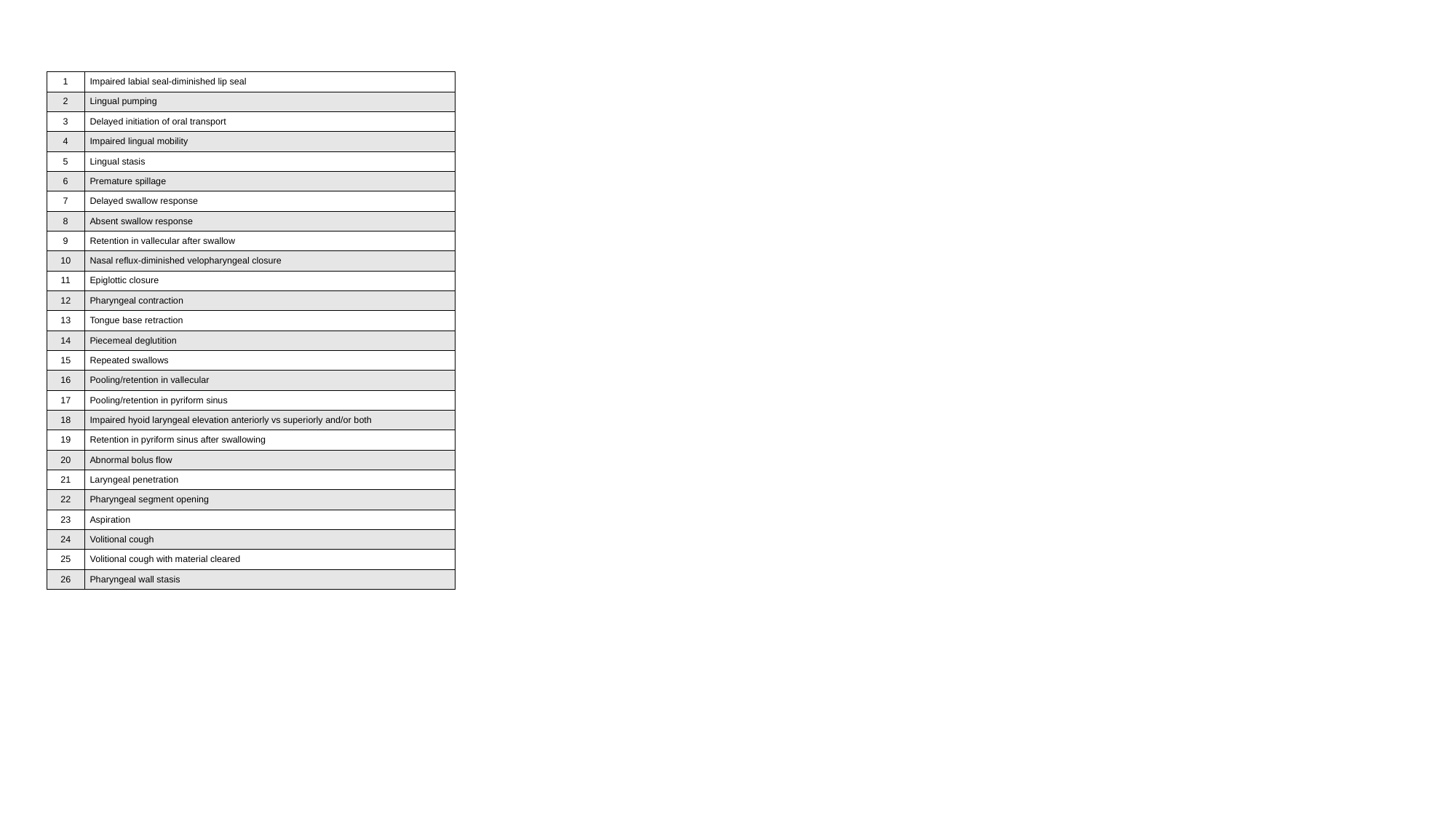

| 1 | Impaired labial seal-diminished lip seal |
| --- | --- |
| 2 | Lingual pumping |
| 3 | Delayed initiation of oral transport |
| 4 | Impaired lingual mobility |
| 5 | Lingual stasis |
| 6 | Premature spillage |
| 7 | Delayed swallow response |
| 8 | Absent swallow response |
| 9 | Retention in vallecular after swallow |
| 10 | Nasal reflux-diminished velopharyngeal closure |
| 11 | Epiglottic closure |
| 12 | Pharyngeal contraction |
| 13 | Tongue base retraction |
| 14 | Piecemeal deglutition |
| 15 | Repeated swallows |
| 16 | Pooling/retention in vallecular |
| 17 | Pooling/retention in pyriform sinus |
| 18 | Impaired hyoid laryngeal elevation anteriorly vs superiorly and/or both |
| 19 | Retention in pyriform sinus after swallowing |
| 20 | Abnormal bolus flow |
| 21 | Laryngeal penetration |
| 22 | Pharyngeal segment opening |
| 23 | Aspiration |
| 24 | Volitional cough |
| 25 | Volitional cough with material cleared |
| 26 | Pharyngeal wall stasis |
